# 1-Cinnamoyltrichilinin from *Melia azedarach* Causes Apoptosis through the p38 MAPK Pathway in HL-60 Human Leukemia Cells

**DOI:** 10.3390/ijms21207506

**Published:** 2020-10-12

**Authors:** Hoibin Jeong, SeonJu Park, Seo-Young Kim, Su-Hyeon Cho, Myeong Seon Jeong, Song-Rae Kim, Jong Bok Seo, Seung Hyun Kim, Kil-Nam Kim

**Affiliations:** 1 Chuncheon Center, Korea Basic Science Institute (KBSI), Chuncheon 24341, Korea; hbj04@kbsi.re.kr (H.J.); sjp19@kbsi.re.kr (S.P.); kimsy11@kbsi.re.kr (S.-Y.K.); chosh93@kbsi.re.kr (S.-H.C.); jms0727@kbsi.re.kr (M.S.J.); ksr87@kbsi.re.kr (S.-R.K.); 2 Seoul Center, Korea Basic Science Institute (KBSI), Seoul 02841, Korea; sjb@kbsi.re.kr; 3 College of Pharmacy, Yonsei Institute of Pharmaceutical Science, Yonsei University, Incheon 21983, Korea

**Keywords:** *Melia azedarach*, natural compound, limonoid, 1-cinnamoyltrichilinin, acute myeloid leukemia, apoptosis, p38 pathway

## Abstract

Acute myeloid leukemia (AML) is an aggressive type of human leukemia with a low survival rate, and its complete remission remains challenging. Although chemotherapy is the first-line treatment of AML, it exerts toxicity in noncancerous cells when used in high doses, thus necessitating the development of novel compounds with a high therapeutic window. This study aimed to investigate the anticancer effects of several compounds derived from the fruits of *Melia azedarach* (a tree with medicinal properties). Among them, 1-cinnamoyltrichilinin (CT) was found to strongly suppress the viability of HL-60 human leukemia cells. CT treatment induced apoptosis and increased nuclear fragmentation and fractional DNA content in HL-60 cells in a dose-dependent manner. CT induced phosphorylation of p38 mitogen-activated protein kinases (p38), though not of c-Jun N-terminal kinase (JNK) and extracellular signal-regulated kinase (ERK), and activated Bcl-2 family proteins towards the proapoptosis and cleavage of caspase-3 and poly (ADP-ribose) polymerase. Both CT-mediated apoptosis and apoptotic protein expression were reversed by treatment with the p38 inhibitor, thereby indicating the p38 pathway to be critical in CT-stimulated apoptosis. The results collectively indicated CT to suppress HL-60 survival by activating the p38 pathway and inducing apoptosis, hence being a novel potential therapeutic agent for AML.

## 1. Introduction

Acute myeloid leukemia (AML) is an aggressive cancer characterized by abnormal proliferation of myeloid cells in the bone marrow or blood [1]. Myeloid progenitor cell differentiation is impaired in AML, thereby disrupting the balance of blood cell populations, resulting in immune dysfunction. As one of the most common leukemias among adults, AML is primarily caused by the accumulation of genetic mutations in hematopoietic stem cells, a progenitor cell type for all blood cells, displaying pervasive aggressiveness in patients with AML, whose five-year overall survival rate is <30% [2]. First-line treatment of AML includes chemotherapy, targeting cell division, which successfully induces recovery [3], since noncancerous cells continue to multiply through cell division; however, the applications are limited in some patients with cancer, especially the older individuals with health risk due to intensive chemotherapy, and only 50% of them achieve complete remission [1]. Improvement of the full-recovery rate would require the screening of new therapeutic compounds with higher efficacy.

Natural products with bioactive components are attractive resources for cancer treatment [4]. They may be extracted from medicinal plants with various therapeutic activities and target multiple functional proteins. Approximately 75% of the compounds currently used in anticancer therapies are associated with natural products [5]. Therefore, screening of the anticancer activity of various natural compounds could be an effective strategy to develop novel agents for cancer treatment.

*Melia azedarach*, a species of deciduous trees commonly grown in the southern region of Korea, has been used as a traditional medicinal herb for centuries [6]. Crude *M. azedarach* leaf extract had earlier been reported to display strong antibacterial activity against *Streptococcus mutans* [7] and radical scavenging properties in cultured cells [8]. Several studies have reported the anticancer effects of *M. azedarach*-derived products until date. Nerome et al. reported extracts from *M. azedarach L.* leaves to inhibit the proliferation of various cancer cell lines and tumor growth in a xenograft mouse model [9]. A methanol extract of *M. azedarach* was reported to exhibit high cytotoxicity in a human breast cancer cell line [10]. These studies suggested the application of *M. azedarach* extract in cancer treatment, encouraging researchers worldwide to identify the specific components in the extract that exert the therapeutic effects in cancer. In the present study, we isolated numerous compounds from the fruits of *M. azedarach* and assessed their antineoplastic activity in human leukemia cells.

## 2. Results

### 2.1. 1-Cinnamoyltrichilinin (CT) Suppressed the Viability of HL-60 Cells

**Figure 1:**
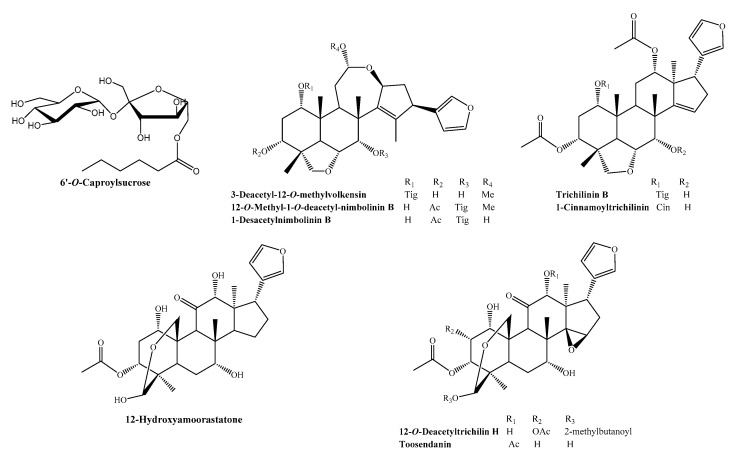
Chemical structures of nine compounds isolated from *Melia azedarach*: 6′-*O*-caproylsucrose, 3-deacetyl-12-*O*-methylvolkensin, 12-*O*-methyl-1-*O*-deacetyl-nimbolinin B, 1-desacetylnimbolinin B, trichilinin B, 1-cinnamoyltrichilinin, 12-hydroxyamoorastatone, 12-*O*-deacetyltrichilin H, and toosendanin.

**Figure 2:**
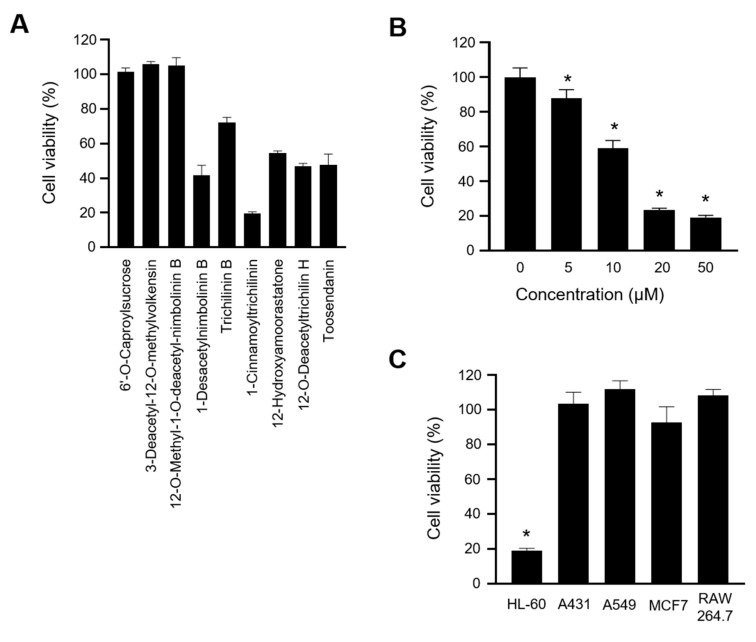
Growth inhibitory effects of 1-cinnamoyltrichilinin (CT) on HL-60 cells. (**A**) 3-(4,5-dimethylthiazol-2-yl)-2,5-diphenyltetrazolium bromide (MTT) colorimetric assay for natural compounds (50 μM) extracted from *M. azedarach*, over 24 h, using HL-60 cells. (**B**) Cell viability of HL-60 cells incubated with various concentrations of CT for 24 h. (**C**) Cytotoxic effects of CT (20 μM) for 24 h on various human cancer cells and a normal cell line (RAW 264.7). Data represent the mean ± SEM from at least triplicate determinations. * indicates *p* < 0.05 calculated by one-way ANOVA.

We successfully isolated nine compounds, namely 6′-*O*-caproylsucrose, 3-deacetyl-12-*O*-methylvolkensin, 12-*O*-methyl-1-*O*-deacetyl-nimbolinin B, 1-desacetylnimbolinin B, trichilinin B, 1-cinnamoyltrichilinin, 12-hydroxyamoorastatone, 12-*O*-deacetyltrichilin H, and toosendanin, from the methanol extracts of *M. azedarach* fruits and determined their structures using high-resolution electrospray ionization mass spectrometry (HR-ESI-MS) and spectroscopic methods, including 1D and 2D nuclear magnetic resonance (NMR) (Figure 1). To identify the cytotoxic compounds in human leukemia, we treated HL-60 cells with the aforementioned compounds and assessed their growth-inhibitory activities. Among the nine compounds, CT significantly decreased cell viability to <20% (Figure 2A) in a dose-dependent manner (Figure 2B). The anticancer properties of CT were effective only in HL-60 cells, not in other human cancer cell lines, such as epidermoid carcinoma (A431), lung epithelial carcinoma (A549), and mammary gland adenocarcinoma (MCF7), at a 20 μM concentration, which is otherwise not toxic to a normal cell line (RAW 264.7) (Figure 2C). These results suggested CT to exert anticancer effects on HL-60 cells in a dose-dependent manner.

### 2.2. CT Induced Apoptosis in HL-60 Cells

**Figure 3:**
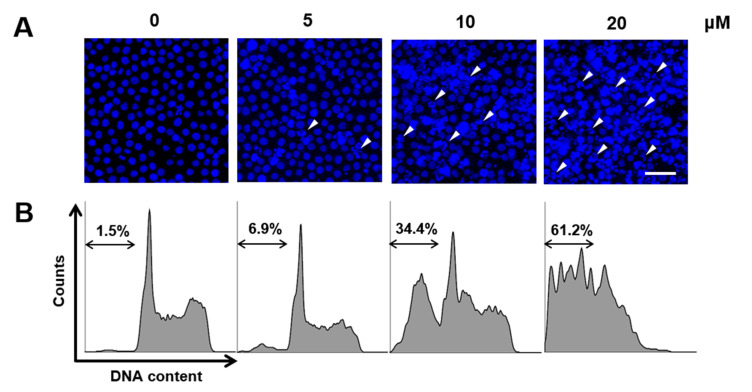
Apoptosis of HL-60 cells mediated by CT. (**A**) Confocal microscopic images showing nuclear staining using Hoechst 33342 and (**B**) flow cytometry using propidium iodide (PI) for measuring HL-60 cells in the sub-G_1_ stage in response to treatment with various concentrations of CT for 24 h. White arrowheads in (**A**) indicate apoptotic bodies. Scale bar indicates 100 μm. Double-sided arrows in flow cytometry data represent the area of the sub-G_1_ stage.

To determine whether CT promotes apoptosis in HL-60 cells and decreases their viability, we performed Hoechst 33342 staining to detect nuclear fragments, which is an indicator of apoptotic progression in cancer cells [11]. As shown in Figure 3A, nuclear fragmentation, including the appearance of apoptotic bodies, was observed in HL-60 cells upon treatment with CT in a dose-dependent manner. Similar results were obtained by assessing the sub-G_1_ population by flow cytometry in response to CT treatment [12]. CT treatment increased the amount of sub-G_1_ DNA content from 1.5% to 61.2% in a dose-dependent manner (Figure 3B), hence suggesting apoptosis in HL-60 cells to be stimulated by CT, thereby decreasing cell viability.

### 2.3. CT Activated p38 Mitogen-Activated Protein Kinase in HL-60 Cells

**Figure 4:**
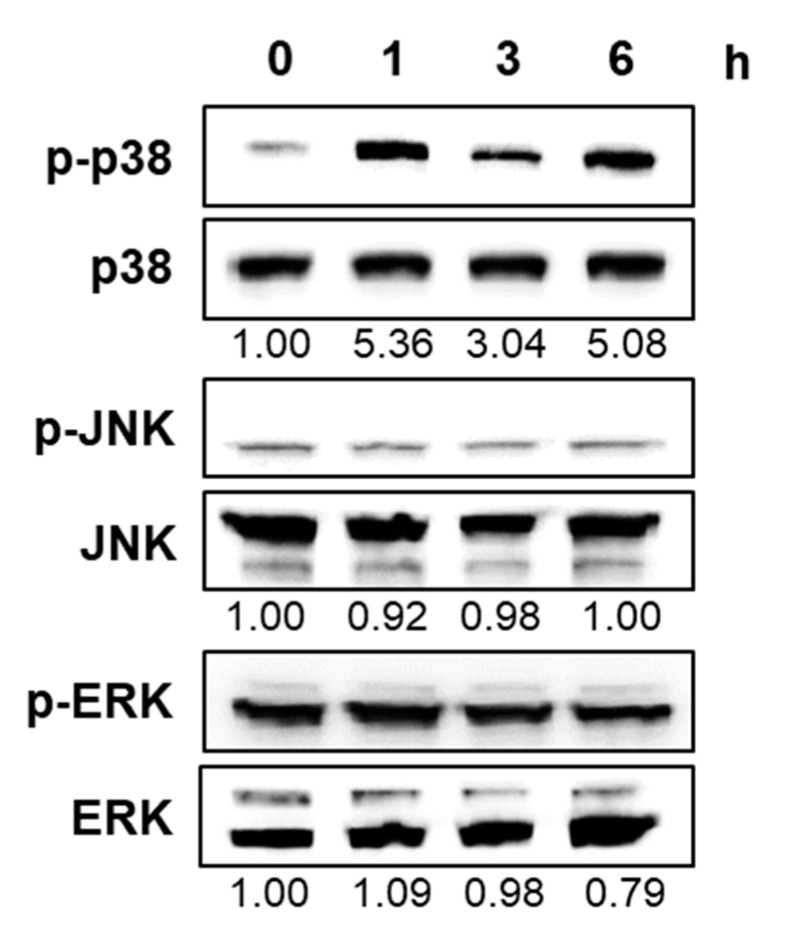
Effect of CT on mitogen-activated protein kinase (MAPK) activation. Western blot using HL-60 cells subjected to CT treatment (10 μM) for different durations to assess MAPKs and their phosphorylated forms, including phospho-p38 (p-p38), p38, phospho- c-Jun N-terminal kinase (JNK) (p-JNK), JNK, phospho- extracellular signal-regulated kinase (ERK) (p-ERK), and ERK. The relative band intensity of the phosphorylated form to the total protein is expressed as the fold change compared to the control (0 h) and is indicated below each band.

Apoptosis is mediated by the activation of mitogen-activated protein kinases (MAPKs), including p38 MAPK (p38), c-Jun N-terminal kinase (JNK), and extracellular signal-regulated kinase (ERK) [13]. To determine the type of MAPK signaling pathway involved in the apoptosis of HL-60 cells exposed to CT, we performed Western blotting for phosphorylated p38, JNK, and ERK in HL-60 cells. Based on the finding of dose-dependent toxicity in HL-60 cells (Figure 2B), we determined 10 μM as the concentration suppressing approximately half the growth of HL-60 cells and used it for monitoring cell growth improvement in subsequent experiments. The phosphorylation level of only p38 was significantly increased upon CT treatment, whereas those of JNK and ERK were not affected significantly (Figure 4); this indicated the selective induction of p38 phosphorylation, though not of JNK or ERK, by CT.

### 2.4. CT-Induced Apoptosis Was Prevented by p38 Inhibition

**Figure 5:**
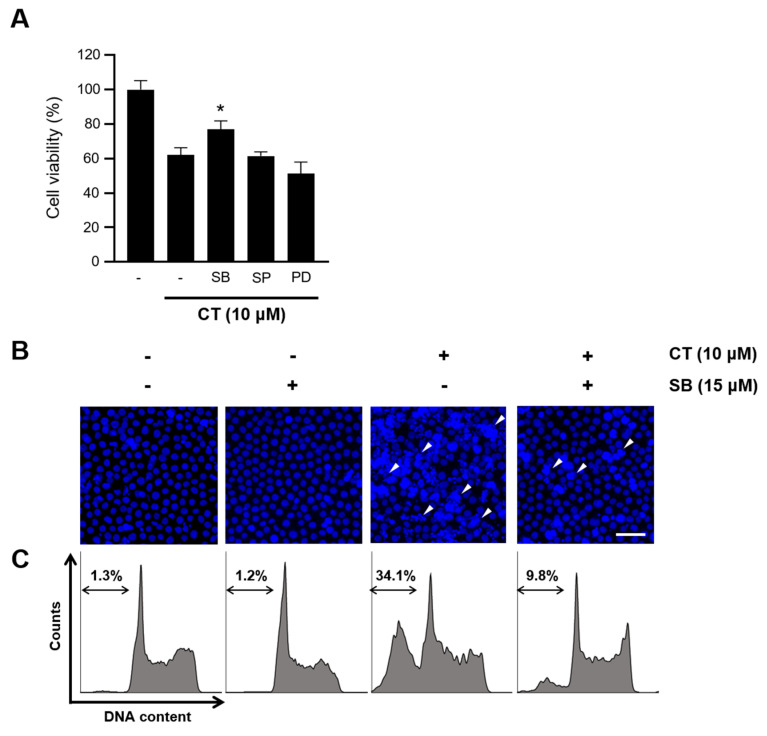
Recovered viability following the inhibition of p38 in CT-treated HL-60 cells. (**A**) MTT assay showing the cytotoxic effects of the CT treatment (10 μM) for 24 h, followed by treatment with MAPK inhibitors for 2 h in HL-60 cells. SB: SB203580 (15 μM), SP: SP600125 (5 μM), and PD: PD98059 (10 μM). * indicates *p* < 0.05 calculated by one-way ANOVA. (**B**) Confocal microscopic images showing nuclear staining with Hoechst 33342 and (**C**) flow cytometry using PI for measuring HL-60 cells in the sub-G_1_ stage following cotreatment with SB203580 (SB) and CT. White arrowheads in the images indicate apoptotic bodies. Scale bar indicates 100 μm. Double-sided arrows in flow cytometry data represent the area of the sub-G_1_ stage.

To evaluate the role of MAPKs in the growth inhibitory effect of CT, HL-60 cells were treated with MAPK inhibitors after CT treatment. As shown in Figure 5A, SP600125 and PD98059, the specific inhibitors of JNK and ERK, respectively, did not influence the reduced viability due to CT treatment; however, SB203580, a specific p38 inhibitor, significantly improved cell viability after CT treatment, thus indicating p38 to be critical for cell death upon treatment with CT. To investigate whether CT-induced apoptosis is hindered by the p38 inhibitor, we performed Hoechst 33342 nuclear staining and flow cytometry. Cotreatment with SB203580 and CT in HL-60 recovered apoptotic bodies (Figure 5B) and remarkably decreased the population of sub-G_1_ cells from 34.1% to 9.8% compared to the CT condition alone (Figure 5C). The results suggested p38 to be critical for the apoptosis of HL-60 cells upon treatment with CT.

### 2.5. Inhibition of p38 Improved HL-60 Viability by Regulating Apoptotic Factors

**Figure 6:**
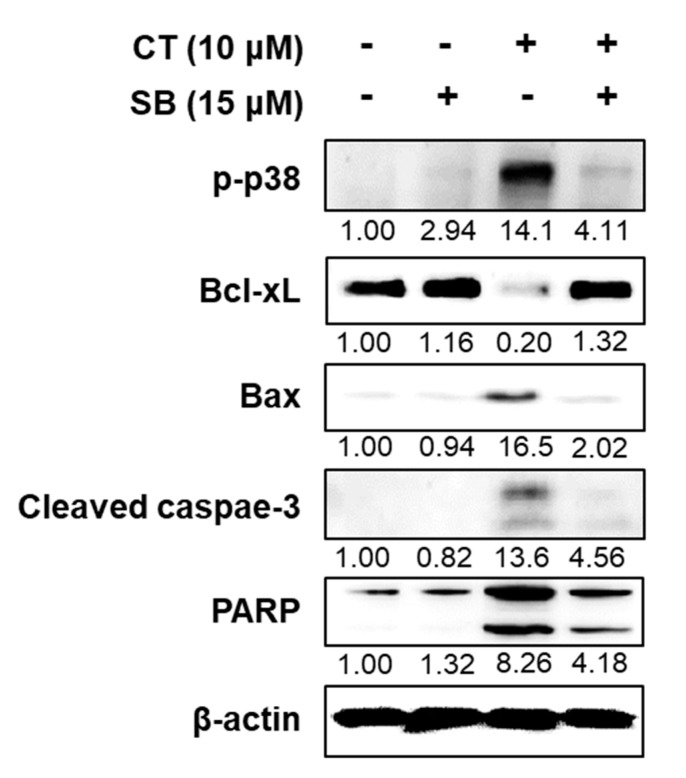
Effect of CT and a p38 inhibitor on apoptotic factors. Western blot using HL-60 cells treated with CT (10 μM) and/or SB203580 (SB, 15 μM) for 6 h to assess phospho-p38 (p-p38) and apoptotic proteins, including Bcl-xL, Bax, cleaved caspase-3, and poly (ADP-ribose) polymerase (PARP). β-Actin was used as the loading control. The relative band intensity of proteins to that of β-actin is expressed as the fold change compared to the control (no treatment) and is indicated below each band.

Apoptosis progresses through the activation of Bcl-2 family protein and cleavage of caspase-3 and poly (ADP-ribose) polymerase (PARP) [14]. To determine the effect of CT on apoptosis, we quantified the expression levels of apoptosis-related proteins, including Bcl-xL (antiapoptotic), Bax (proapoptotic), cleaved caspase-3, and PARP, using Western blotting. First, p38 MAPK phosphorylation, enhanced by CT, was recovered after cotreatment with SB203580 (Figure 6). Next, CT decreased Bcl-xL and increased Bax, cleaved caspase-3, and cleaved PARP, thus activating proapoptotic factors; however, SB203580 partially or completely reversed the expression of all proteins to the baseline levels (Figure 6). These results suggested the activation of apoptotic signaling cascades in HL-60 cells by CT via p38 phosphorylation.

## 3. Discussion

This study provided clear evidence of CT inducing apoptosis in HL-60 cells via p38 phosphorylation. CT is an oxygenated triterpenoid derivative containing a furan ring [15]. Limonoids, identified as the core component causing bitterness in citrus fruits, exhibit anticancer activity both in vitro and in vivo [16,17]. In neuroblastoma cells, bioactive limonoid compounds induce apoptosis by increasing caspase-3/7 activity [16]. Limonoids from citrus fruits have been reported to suppress chemically induced hepatocarcinogenesis in rats by decreasing lipid peroxidation and oxidative stress [17]. Some limonoid compounds isolated from *M. azedarach* have been shown to induce cell cycle arrest and apoptosis in human leukemia cells via ERK1/2 activation [18]. The present results suggested a trichilin-type limonoid to trigger apoptosis and hinder cell growth in cancer cells.

Apoptosis is a type of programmed cell death that maintains tissue homeostasis during aging and development [11]. During apoptosis, cells shrink and display nuclear and organellar lysis, contrary to necrosis, in which they undergo energy-independent cell death, accompanied by swelling. Necrosis is characterized as an accidental cell death, with an uncontrolled release of intracellular contents, thus initiating inflammatory responses in surrounding tissues [11]. Thus, examining therapeutic compounds inducing apoptosis rather than necrosis is considered an appropriate strategy in cancer research. Here, we treated HL-60 cells with CT and examined whether it triggers apoptosis in these cells. The breakdown of cellular components yields small membrane-bound compartments containing DNA, called apoptotic bodies, which are detectable through nuclear staining [11]. DNA multimers of <200 bp are formed, owing to the extracellular leakage of apoptotic nuclear fragments, thus reducing the overall DNA content; such cells are referred to as sub-G_1_ cells, and confirmed by flow cytometry [12]. Cancer cells evade apoptosis via defective apoptotic signaling due to accumulated mutations [19]. Half of all tumor types present overexpressed antiapoptotic Bcl-2 proteins and the loss of proapoptotic factors, resulting in resistance to apoptotic stimuli, including anticancer therapeutic drugs [20]. One method of treating tumors is to terminate their uncontrollable growth and activate apoptosis. Targeting apoptotic factors is an attractive method for inducing cell death in tumors. In this study, CT significantly increased nuclear fragmentation and sub-G_1_ DNA content and triggered apoptosis in HL-60 cells.

MAPK proteins, key regulators of cellular proliferation, differentiation, and apoptosis, are activated by cytokines or extracellular stresses [13]. Upon stimulation, serine/threonine kinases are phosphorylated and regulate numerous substrates, leading to differential signal transduction, depending on the type of stimulus. The three primary components of MAPKs are p38, JNK, and ERK; ERK is primarily involved in survival, whereas p38 and JNK are responsive to stress conditions, including the apoptotic phenotype [21]. In cancer cells, however, MAPK signaling cascades are dysregulated, owing to genetic mutations or environmental stimuli; hence, numerous studies have focused on developing therapeutic agents to restore the balance of MAPK function [22]. In this study, p38, though not JNK and ERK, was phosphorylated during CT-mediated HL-60 apoptosis. To evaluate the direct association of the MAPK pathway in cancer apoptosis, many researchers have determined whether MAPK inhibitors can reverse compound-mediated apoptosis. We observed that the p38 inhibitor restored the elevated levels of apoptotic bodies and sub-G_1_ DNA content to the baseline levels, whereas JNK and ERK inhibitors had no such effect, suggesting p38 to play an important role in the apoptosis of HL-60 cells upon CT treatment.

The apoptotic pathway is triggered by mitochondrial cytochrome *c* release [23]. Once released, cytochrome *c* binds to apoptotic protease activating factor-1, generating an apoptosome, which, in turn, initiates the caspase cascade, cleaving pro-caspase-9 and pro-caspase-3 to their active forms, thereby leading to PARP cleavage [14]. Mitochondrial cytochrome *c* release is mediated by Bcl-2 family proteins. Under physiological conditions, proapoptotic proteins, including Bax, Bak, and Bad, are downregulated. In the presence of apoptotic signals, however, the ratio of proapoptotic to prosurvival proteins, including Bcl-xL and Bcl-2, is increased, resulting in the permeabilization of the mitochondrial outer membrane and the release of cytochrome *c* [23]. The present results showed CT to upregulate Bax, cleaved caspase-3, and PARP and downregulate Bcl-xL, implying the apoptotic caspase cascade to be triggered by CT. This activation could be reversed by the p38 inhibitor, hence validating the essential role of p38 activation in CT-induced apoptosis in HL-60 cells.

In summary, this study demonstrated the anticancer activity of CT in HL-60 human leukemia. CT induced apoptosis in HL-60 cells by upregulating apoptotic proteins, including Bax, caspase-3, and PARP. Mechanistically, p38 activation was demonstrated to be important for CT-mediated apoptosis, which could be prevented by the p38 inhibitor. The results together suggested CT to be a potent anticancer therapeutic agent, owing to its ability to induce p38 phosphorylation and apoptosis via Bcl-xL, Bax, and caspase-3. To investigate how CT activates p38 and specifically affects human leukemia cells, we plan to perform further mechanistic studies. We believe that our findings would be valuable as fundamental data for developing CT as a novel therapeutic agent to treat AML.

## 4. Materials and Methods

### 4.1. Materials

Rowell Park Memorial Institute (RPMI)-1640 medium was purchased from Hyclone Laboratories Inc. (Logan, UT, USA). Dulbecco’s modified Eagle’s medium (DMEM) was purchased from Welgene (Gyeongsangbuk-do, Korea). Fetal bovine serum (FBS) was purchased from Omega Scientific, Inc. (Tarzana, CA, USA). Penicillin and streptomycin were purchased from Invitrogen (Carlsbad, CA, USA). SB203580, SP600125, and PD98059 were purchased from Calbiochem (San Diego, CA, USA). 3-(4,5-dimethylthiazol-2-yl)-2,5-diphenyltetrazolium bromide (MTT), dimethyl sulfoxide (DMSO), Hoechst 33342, propidium iodide (PI), RNase A, and RIPA buffer were purchased from Sigma-Aldrich (St. Louis, MO, USA). EDTA solution was procured from Bioneer (Dajgeon-si, Korea). NuPAGE 4–12% Bis-Tris gel was purchased from Life Technology (Carlsbad, CA, USA). Polyvinylidene fluoride membranes were purchased from Bio-Rad Laboratories (Richmond, CA, USA). Anti-human phospho-p38 (Thr180/Thy182, Cat# 9211), rabbit anti-human p38 (Cat# 9212), rabbit anti-human phospho-JNK (Thr183/Tyr185, Cat#9251), rabbit anti-human JNK (Cat# 9252), rabbit anti-human phospho-ERK (Thr202/Tyr204, Cat#9101), rabbit anti-human ERK (Cat# 9102), rabbit anti-human Bcl-xL (Cat# 2762), rabbit anti-human Bax (Cat# 2772), rabbit anti-human cleaved caspase-3 (Cat# 9664), rabbit anti-human PARP (Car# 9542), goat anti-rabbit IgG (Cat# 7074), and goat anti-mouse IgG (Cat# 7076) were purchased from Cell Signaling Technology (Danvers, MA, USA). Mouse anti-human β-actin was purchased from Santa Cruz Biotechnology (Dallas, TX, USA).

### 4.2. Plant Material

Fruits of *M. azedarach* were harvested in Hwaseong, Gyeonggi, Korea in January 2017 and authenticated by Dr. Rack-Seon Seong, Director, Center of Natural Resources Research, Jeonnam Bioindustry Foundation. A voucher specimen (MT201701) was deposited at the Herbarium of the College of Pharmacy, Yonsei Institute of Pharmaceutical Sciences, Yonsei University, Incheon, Korea.

### 4.3. Extraction and Isolation

Dried *M. azedarach* fruits (3.0 kg) were extracted with MeOH (3 × 10 L, 50 °C) and sonicated for 4 h to obtain a 400.0-g extract after solvent evaporation. This extract was suspended in H_2_O and successively partitioned with CHCl_3_ and EtOAc to obtain CHCl_3_ (MT1), EtOAc (MT2), and H_2_O (MT3) extracts after removal of the solvents in vacuo. MT1 was subjected to silica gel column chromatography (CC) and eluted against a gradient of *n*-hexane:acetone (10:1 to 1:1, *v*/*v*) to obtain four subfractions: MT1A, MT1B, MT1C, and MT1D. MT1C was chromatographed on a silica gel column and eluted with CHCl_3_:acetone (1:1, *v*/*v*), yielding three smaller fractions: MT1C1–MT1C3. MT1C1 was again chromatographed by reverse-phase (RP)-18 CC, eluting with MeOH: H2O (2:1, *v*/*v*) to obtain toosendanin. The MT1D fraction was chromatographed on a silica gel column and eluted with *n*-hexane: EtOAc (1:1, *v*/*v*) to obtain five smaller fractions: MT1D1–MT1D5. MT1D3 was further chromatographed through high-performance liquid chromatography (HPLC) using a J’sphere ODS H-80 (YMC Co. Ltd., Wilmington, NC, USA), 250 mm × 20 mm column, 40% aqueous MeCN at a flow rate of 3 mL/min to yield 3-deacetyl-12-*O*-methylvolkensin, 12-*O*-methyl-1-O-deacetyl-nimbolinin B, 1-desacetylnimbolinin B, trichilinin B, and 1-cinnamoyltrichilinin. MT1D4 chromatographed via HPLC, using 50% aqueous MeCN, yielded 6′-*O*-caproylsucrose, 12-hydroxyamoorastatone, and 12-*O*-deacetyltrichilin H. Their structures were determined by HR-ESI-MS analysis and spectroscopic methods, including 1D and 2D NMR [24,25,26,27,28,29]. The tested constituents were obtained at purities >95%, as revealed by HPLC.

### 4.4. Cell Culture and Materials

HL-60 (a human AML cell line), A431 (a human epidermoid carcinoma cell line), A549 (a human lung epithelial carcinoma cell line), and MCF7 cells (a human mammary gland adenocarcinoma cell line) were cultured in RPMI-1640 medium, and RAW264.7 cells (a murine macrophage cell line) were cultured in DMEM supplemented with 10% FBS, penicillin (100 U/mL), and streptomycin (100 μg/mL). All cell lines were purchased from ATCC (Manassas, VA, USA) and maintained at 37 °C in a 5% CO_2_ incubator.

### 4.5. Cell Growth Inhibition Assays

Cytotoxicity of the isolated or extracted compounds was assessed through a colorimetric assay. Approximately 1 × 10^5^ cells/mL were seeded in 96-well plates and incubated with the compounds for 24 h. MAPK inhibitors were administered 2 h before the compounds were assessed. Thereafter, 100-μg/mL MTT was added to each well. After 2.5-h incubation at 37 °C, the supernatants were aspirated, and cells were treated with DMSO to dissolve the formazan crystals. Absorbance of the colored solution was determined at 540 nm using a SpectraMax M2/M2e (Molecular Devices, San Jose, CA, USA).

### 4.6. Nuclear Staining with Hoechst 33342

HL-60 cells were seeded in 96-well plates at 1 × 10^5^ cells/mL. Compounds with or without SB203580 were added to the cells. After 24 h of incubation, 10 μg/mL of Hoechst 33342 was added to each well, followed by 10 min of incubation at 37 °C. The stained cells were observed under an LSM880 confocal microscope (Carl Zeiss, Oberkochen, Germany) to assess the degree of nuclear condensation.

### 4.7. Sub-G_1_ Population Measurement by Flow Cytometry

In total, 1 × 10^5^ HL-60 cells/mL were seeded in 60-mm culture plates. Compounds with or without SB203580 were added to them. After 24 h of incubation, the cells were harvested and fixed in 1 mL of 70% cold ethanol for 30 min at 4 °C. The cells were then washed twice with 2-mM EDTA-containing PBS and incubated with 1 mL of 2-mM EDTA-PBS containing 100-μg/mL PI and 100-μg/mL RNase A for 30 min at 37 °C. Samples were introduced into BD LSRFortessa-X20™ (BD, Franklin Lakes, NJ, USA) for subsequent analysis.

### 4.8. Western Blot

Cell lysates were prepared in RIPA buffer. Quantified protein lysates were loaded into NuPAGE 4–12% Bis-Tris gels, which were then blotted onto a polyvinylidene fluoride membrane. Primary antibodies, including rabbit anti-human phospho-p38, rabbit anti-human p38, rabbit anti-human phospho-JNK, rabbit anti-human JNK, rabbit anti-human phospho-ERK, rabbit anti-human ERK, rabbit anti-human Bcl-xL, rabbit anti-human Bax, rabbit anti-human cleaved caspase-3, rabbit anti-human PARP, and mouse anti-human β-actin antibodies, were diluted at 1:1000 and incubated overnight at 4 °C. Secondary antibodies, including goat anti-rabbit IgG and goat anti-mouse IgG, were diluted at 1:3000 and incubated for 1.5 h at 25 °C. Signals were developed using the SuperSignal West Femto Trial Kit (Thermo Fisher Scientific, Waltham, MA, USA), and images were acquired using Fusion FX (Vilber Lourmat Ste, Collegien, France). Band intensity was quantified using ImageJ software (National Institutes of Health, Bethesda, MD, USA).

### 4.9. Statistical Analysis

Data were statistically compared by two-tailed one-way ANOVA and Tukey’s post-test using Prism software (Version 4.00; GraphPad Inc., La Jolla, CA, USA). Data were considered statistically significant at *p* < 0.05.

## Abbreviations

AML	Acute myeloid leukemia
CT	1-Cinnamoyltrichilinin
HR-ESI-MS	High-resolution electrospray ionization mass spectroscopy
NMR	Nuclear magnetic resonance
MAPK	Mitogen-activated protein kinase
JNK	c-Jun-N-terminal kinase
ERK	Extracellular signal-regulated kinase
PARP	Poly (ADP-ribose) polymerase
MTT	3-(4,5-dimethylthiazol-2-yl)-2,5-diphenyltetrazolium bromide
PI	Propidium iodide
CC	Column chromatography
HPLC	High-performance liquid chromatography
